# The cross-sectional study on the association between physical exercise and restrained eating among Chinese college students: the chain mediating roles of emotional intelligence and perceived stress

**DOI:** 10.3389/fpsyg.2026.1805606

**Published:** 2026-04-14

**Authors:** Qiang Guo, Tong Liu, Zhonggen Yin, Mingda Li

**Affiliations:** School of Sports Training, Chengdu Sport University, Chengdu, China

**Keywords:** college students, emotional intelligence, perceived stress, physical exercise, restrained eating

## Abstract

**Background:**

Restrained eating, a dietary pattern with potential health risks, is prevalent among college students. Although physical exercise is associated with restrained eating, the underlying psychological mechanisms remain unclear. This study examined the relationship between physical exercise and restrained eating among Chinese university students, focusing on the chain mediating roles of emotional intelligence (EI) and perceived stress.

**Methods:**

Participants were 1,640 college students (58.5% male; mean age 19.29 ± 1.16 years) from eight provinces in China. They completed measures of physical exercise [Physical Activity Rating Scale-3 (PARS-3)], restrained eating [Dutch Eating Behavior Questionnaire (DEBQ)], emotional intelligence [Wong and Law Emotional Intelligence Scale (WLEIS)], and perceived stress [Perceived Stress Scale (PSS)]. Mediation analysis was conducted using SPSS 26.0 with the PROCESS macro (Model 6) and 5,000 bootstrap samples.

**Results:**

Physical exercise was negatively correlated with restrained eating (*r* = −0.369, *p* < 0.001). Regression analyses showed that physical exercise (β = −0.406, *p* < 0.001) and emotional intelligence (β = −0.337, *p* < 0.001) negatively predicted restrained eating, while perceived stress positively predicted it (β = 0.332, *p* < 0.001). Mediation analyses revealed significant indirect effects through emotional intelligence (indirect effect = −0.023, 95% *CI* [−0.030, −0.017]), perceived stress (indirect effect = −0.019, 95% *CI* [−0.025, −0.013]), and the sequential pathway through both mediators (indirect effect = −0.002, 95% *CI* [−0.004, −0.001]). The total indirect effect (−0.044) accounted for 35.8% of the total effect (−0.123).

**Conclusion:**

Physical exercise is negatively associated with restrained eating among Chinese college students, with emotional intelligence and perceived stress serving as independent and sequential mediators. These findings highlight the importance of integrating emotional competence and stress management into physical activity interventions to promote healthy eating behaviors in university populations.

## 1 Introduction

Restrained eating, first conceptualized by Herman (1978), refers to the chronic, intentional restriction of food intake aimed at controlling or reducing body weight. Although commonly viewed as a weight management strategy, accumulating evidence suggests that restrained eating is associated with a range of maladaptive outcomes, including eating disorders (ED), binge eating, and body dissatisfaction (Bourne et al., 1998; Johnson and Wardle, 2005; Spoor et al., 2006). It is important to distinguish restrained eating from clinically diagnosed eating disorders such as anorexia nervosa and bulimia nervosa as defined in the DSM-5 (American Psychiatric Association, 2013). Unlike these clinical conditions, restrained eating represents a behavioral pattern rather than a psychiatric diagnosis. Nevertheless, persistent and extreme forms of restrained eating are considered significant risk factors and subclinical manifestations of eating disorders (Johnson and Wardle, 2005). Accordingly, the present study focuses on restrained eating tendencies within non-clinical populations, rather than on clinical eating disorder diagnoses.

Restrained eating behaviors are particularly prevalent among college students. A survey of Chinese university students found that 52.8% had attempted to lose weight through dietary restriction (Yong et al., 2021). The college years represent a critical developmental period marked by significant lifestyle transitions that can shape long-term health behaviors (Montgomery and Côté, 2003). Within sociocultural contexts that emphasize thinness and idealized body shapes, many students adopt dietary restraint as a weight management strategy (Grogan, 2021). However, such practices are often associated with negative outcomes, including body dissatisfaction and unhealthy compensatory behaviors such as prolonged fasting or self-induced vomiting (Schaumberg et al., 2014; Shi et al., 2023). Moreover, restrained eating may have broader behavioral consequences. For instance, a study by Pan et al. (2025) demonstrated that restrained eating among Chinese college students was positively associated with exercise addiction, a relationship serially mediated by social physique anxiety and grit. These findings suggest that restrained eating not only serves as a potential precursor to eating disorders but may also be linked to other maladaptive behavioral patterns. Concurrently, declining physical fitness and increasing unhealthy lifestyle behaviors among college students have raised growing public health concern (Almoraie et al., 2024). Physical exercise is widely recognized as a key health-promoting behavior and a modifiable lifestyle factor that may influence eating behaviors (Byrne et al., 2016). Regular physical activity has been associated with healthier dietary patterns and improved psychological well-being (Mikkelsen et al., 2017; Xu et al., 2019). For individuals seeking to manage weight or improve body image, engaging in physical activity may offer a healthier alternative to extreme dietary restriction (Teixeira et al., 2006). Consistently, Hu et al. (2025) reported that physical exercise exerted a buffering effect on the relationship between social appearance anxiety and restrained eating among Chinese female college students. highlighting the potential protective role of exercise in this context.

However, the relationship between physical exercise and restrained eating appears complex and inconsistent across studies. On one hand, exercise may is associated with lower restrained eating by enhancing body satisfaction and self-regulatory capacity; on the other, it may reinforce dietary restraint when physical activity is primarily motivated by weight- or appearance-related concerns (Annesi, 2019; Schultchen et al., 2019). Illustrating this complexity, Ugraş et al. (2025) found that among Turkish university students, positive attitudes toward physical activity were associated with lower body dissatisfaction, binge eating, and purging, but were not significantly related to restrained eating. These findings suggest that the relationship between physical exercise and restrained eating may be indirect, operating through underlying psychological mechanisms.

Psychological factors are likely central to this relationship. Emotional intelligence (EI)—defined as the ability to perceive, understand, and regulate emotions—has been associated with healthier coping strategies and a is associated with lowerd risk of maladaptive eating behaviors (Romero-Mesa et al., 2021). Individuals with higher emotional intelligence are better equipped to regulate emotional responses and adopt adaptive coping strategies, rather than resorting to restrictive eating (Filaire et al., 2011). Conversely, perceived stress has been consistently linked to unhealthy dietary patterns and disordered eating tendencies among college students (Al-Asadi, 2014; Choi, 2020). Importantly, emotional intelligence may also facilitate more effective stress management, thereby reducing the likelihood of maladaptive eating behaviors (Romero-Mesa et al., 2021). Taken together, these lines of evidence suggest that emotional intelligence and perceived stress may serve as important mediators in the relationship between physical exercise and restrained eating. Accordingly, the present study aimed to examine the association between physical exercise and restrained eating among Chinese college students, with a specific focus on the potential chain mediating roles of emotional intelligence and perceived stress.

## 2 Literature review and hypotheses

### 2.1 Physical exercise and restrained eating

Restrained eating is often considered a prominent subclinical manifestation of eating disorders (ED). Eating disorders are not merely detrimental to physical health; they constitute a form of mental illness that exerts profoundly negative effects on both psychological and physical well-being (Doll et al., 2005). Given the conceptual overlap among eating-related constructs, it is important to distinguish restrained eating from other related but distinct concepts. Mindful eating refers to a non-judgmental awareness of physical and emotional sensations while eating, emphasizing attention to the present moment without reaction (Hussain et al., 2025). Intuitive eating involves eating in response to physiological hunger and satiety cues rather than emotional or external triggers, and is characterized by unconditional permission to eat and reliance on internal cues (Kerin et al., 2019). In contrast, restrained eating, the focus of the present study, involves a conscious and cognitive effort to restrict food intake for weight control, often overriding internal hunger cues (Polivy and Herman, 1985). While mindful and intuitive eating represent adaptive, internally regulated eating styles, restrained eating reflects a maladaptive, cognitively driven pattern. Accordingly, this study focuses exclusively on restrained eating as the outcome variable. Subclinical eating disorder manifestations primarily involve dieting and bulimic behaviors. While clinical eating disorders are relatively uncommon, subclinical symptoms are highly prevalent, affecting approximately 60% of girls and 30% of boys, with evidence suggesting a continued upward trend (McClelland et al., 2020). Restrained eating behaviors are particularly common among young people, especially female college students. Research indicates that approximately 53.5% of college students engage in weight-loss behaviors to varying degrees (Van Dyne et al., 2023). Paradoxically, restrained eating, the most prevalent weight-loss method, often fails to achieve its intended purpose. Over time, dietary restriction can trigger binge eating, ultimately leading to weight gain (Casari et al., 2026). This creates a self-perpetuating cycle in which increased restraint begets increased disinhibition. Such repeated cycles of restrained eating and binge eating readily precipitate negative emotional states, including low self-esteem and anxiety (Burton and Abbott, 2019), and in severe cases, may even contribute to suicidal behavior (Wang S. B. et al., 2020). Given these detrimental consequences, it is essential to investigate the antecedents of restrained eating and identify potential mechanisms for effective intervention.

Physical exercise is considered one of the most significant and modifiable health behaviors among college students, with the potential to play an important role in regulating dietary behavior (Cook et al., 2016). According to Self-Regulation Theory (Baumeister, 1997), which posits that behavioral control skills can transfer across domains, physical exercise may reflect—and further cultivate—an individual's capacity for self-regulation that extends to dietary control (Johnson et al., 2012). Specifically, regular physical activity has been shown to alleviate psychological stress and enhance emotional regulation efficiency (Bernstein and McNally, 2018), thereby reducing the individual's reliance on excessive dietary control as a means of managing weight or emotions. When self-regulatory capacity is primarily channeled through exercise behavior, the need for strict dietary restrictions diminishes, manifesting as is associated with lowered engagement in restrained eating patterns (Johnson et al., 2012). Based on this theoretical framework, we propose the following hypothesis:

**H1:** Physical exercise is negatively associated with restrained eating among college students.

### 2.2 The mediating role of emotional intelligence

Emotional intelligence (EI), defined as the capacity to perceive, regulate, and use emotions, is widely recognized as a key determinant of psychological well-being (Salovey and Mayer, 1990). During the university years, students navigate a delicate transitional phase between adolescence and adulthood, grappling with interconnected challenges related to academic pressure, interpersonal relationships, and identity formation (Mulaudzi, 2023). These developmental stressors render college students particularly vulnerable to emotional dysregulation and psychological distress when confronting setbacks or failures (Granieri et al., 2021). Consequently, enhancing emotional intelligence is regarded as a crucial pathway for promoting mental well-being and adaptive development in this population (Savchuk et al., 2022). Although emotional intelligence has been conceptualized both as a cognitive ability (Elfenbein and MacCann, 2017) and as a personality trait (Petrides et al., 2018), researchers broadly agree that it plays a fundamental role in navigating real-life challenges and achieving personal success (Ayeni et al., 2024; Pashchenko et al., 2024; Andres, 2025). Deficits in emotional intelligence have been linked to eating disorders and other impulsive behaviors (Romero-Mesa et al., 2021). Moreover, emotional and social cognitive abilities—along with eating disorder symptoms, exhibit developmental trajectories that evolve across the lifespan (Romero-Mesa et al., 2021). For instance, research on the dietary attitudes of judo athletes has highlighted the relevance of emotional intelligence in the context of disordered eating (Filaire et al., 2011).

Grounded in Emotion Regulation Theory (Salovey and Mayer, 1990), which posits that individuals with higher emotional intelligence are better equipped to manage negative emotions without resorting to maladaptive behaviors, we propose that emotional intelligence serves as a key mechanism linking physical exercise to restrained eating. Individuals with higher emotional intelligence are more adept at accurately identifying, comprehending, and regulating their emotional responses. This capacity is associated with lowers the likelihood of turning to restrained eating as a coping strategy for negative emotions or perceived loss of control (Foye et al., 2019). Conversely, individuals with lower emotional intelligence may be more inclined to employ dietary restriction as an alternative means of emotion regulation, thereby increasing their risk of engaging in restrained eating behaviors. Physical exercise has been shown to enhance emotional awareness and regulation capacity (Gáspár et al., 2017). Accordingly, emotional intelligence may constitute a crucial psychological pathway through which physical exercise influences restrained eating. Based on this rationale, we propose the following hypothesis:

**H2**: Emotional intelligence mediates the relationship between physical exercise and restrained eating among college students.

### 2.3 The mediating role of perceived stress

Perceived stress refers to an individual's subjective appraisal of the general pressures encountered in daily life and their perceived capacity to cope with such demands (Bartlett, 1998). As a significant external trigger for restrained eating, heightened perceived stress has been consistently associated with a range of adverse health outcomes. Prolonged exposure to stress not only undermines mental well-being but may also disrupt daily behavioral patterns. Existing research indicates that perceived stress plays a crucial role in the development of restrained eating and related disordered eating behaviors (Joseph et al., 2018). Over recent decades, researchers have increasingly recognized that individuals with eating disorders often exhibit heightened emotional reactivity in their attitudes toward food and their bodies (McNamara et al., 2008). Consequently, emotions have emerged as a central focus in the study of restrained eating (Snoek et al., 2013; Godet et al., 2022). Previous research has shown that individuals with eating disorders frequently resort to disordered eating patterns as a maladaptive substitute when they are unable to manage negative emotions effectively (Dingemans et al., 2017). Social media pressure has also been implicated as a contributing factor to dieting behaviors (Saade et al., 2019). More broadly, individuals who lack the capacity to regulate negative emotions may turn to alternative strategies, such as alcohol abuse or binge eating, to alleviate or escape stress-induced distress. However, these approaches typically undermine self-regulatory capacity and can be detrimental to long-term health (Hagger et al., 2009). Thus, it is plausible that individuals exhibiting disordered eating behaviors may experience deficits in emotion regulation (Prefit and Szentagotai-Tătar, 2018).

Drawing on Stress and Coping Theory (Lazarus and Folkman, 1984), which posits that individuals under stress adopt controllable behaviors to restore a sense of control, we propose that perceived stress serves as a key mechanism linking physical exercise to restrained eating. Heightened perceived stress increases psychological strain and diminishes adaptive coping capacities. Under such conditions, individuals are more likely to turn to restrained eating as a controllable and immediate coping strategy to regain a sense of control over their physical or emotional state. Consequently, higher levels of perceived stress are associated with a greater likelihood of engaging in restrained eating behaviors. Physical exercise has been widely recognized as an effective means of alleviating stress and improving psychological well-being (Gerber and Pühse, 2009). As such, perceived stress may represent a significant psychological pathway through which physical exercise influences restrained eating. Based on this rationale, we propose the following hypothesis:

**H3:** Perceived stress mediates the relationship between physical exercise and restrained eating among college students.

### 2.4 The relationship between physical exercise, emotional intelligence, perceived stress, and restrained eating

Research has demonstrated that physical exercise, as an external intervention, can exert an effect on an individual's restrained eating behavior (Andrade et al., 2010), Physical activity is closely linked to cognition (Erickson et al., 2015). Research has found that women who are more preoccupied with their body image experience heightened anxiety about their appearance after exercise and report greater levels of body dissatisfaction (Davis, 1990). Activity-based models of anorexia nervosa and fundamental research in exercise physiology indicate that moderate to vigorous physical activity leads to a reduction in energy intake relative to metabolic demands (Forney et al., 2025).

Integrating Compensatory Control Theory (Kay et al., 2008), with the aforementioned theoretical frameworks, we propose that individuals experiencing a subjectively perceived lack of control are motivated to restore psychological order and certainty through predictable, controllable behaviors. Physical exercise and emotional intelligence enhance one's sense of mastery over bodily states and emotional experiences, thereby reducing perceived stress. Conversely, when perceived stress is elevated and subjective control is undermined, restrained eating, as a highly structured and immediately controllable behavior, may be adopted as a compensatory strategy to regain a sense of control. Thus, engaging in physical exercise and cultivating emotional intelligence may enhance perceived control, is associated with lower stress perception, and indirectly mitigate restrained eating, whereas heightened perceived stress may reinforce reliance on such maladaptive eating patterns.

Specifically, as physical activity levels increase, the association between emotional intelligence and perceived stress may become progressively stronger, thereby indirectly influencing dietary behavior. These findings suggest that physical exercise does not exert a uniform protective effect; rather, it may interact with emotional and stress-related processes in more nuanced ways (Stults-Kolehmainen and Sinha, 2014). Against this theoretical backdrop, physical exercise may be associated with dietary restraint either directly or indirectly through emotional intelligence and perceived stress. For instance, physical exercise may enhance emotional intelligence (Zysberg and Hemmel, 2018), which in turn may is associated with lower perceived stress, thereby decreasing the likelihood of restrained eating behaviors. Consistent with this view, evidence suggests that emotional intelligence is closely linked to effective stress management (Matthews et al., 2017). Furthermore, variations in physical activity levels may moderate the strength of the association between emotional intelligence and perceived stress (Castro-Sánchez et al., 2022), contributing to individual differences in diet-related outcomes. In summary, we propose the following hypothesis:

**H4:** Emotional intelligence and perceived stress exert a chain mediating effect between physical exercise and restrained eating among college students.

This study integrates four theoretical frameworks—Self-Regulation Theory, Emotion Regulation Theory, Stress and Coping Theory, and Compensatory Control Theory—to elucidate the relationship between physical exercise and restrained eating. According to Self-Regulation Theory (Baumeister, 1997), physical exercise reflects an individual's capacity for behavioral control, a capacity that can be extended to dietary behavior, manifesting as is associated with lowered restrained eating. Emotion Regulation Theory (Salovey and Mayer, 1990) suggests that individuals with higher emotional intelligence are better able to identify and manage negative emotions, thereby diminishing the need to rely on restrained eating as a maladaptive coping mechanism. Stress and Coping Theory (Lazarus and Folkman, 1984) posits that individuals experiencing elevated stress tend to adopt immediate, controllable behaviors such as dietary restriction to restore a sense of control. Finally, Compensatory Control Theory (Kay et al., 2008) proposes that individuals who perceive a lack of control are motivated to restore psychological order and certainty through predictable, controllable behaviors. Building on these interconnected frameworks, the present study constructs a chain mediation model hypothesizing that physical exercise is positively associated with emotional intelligence, which in turn is negatively associated with perceived stress, and that perceived stress is positively associated with restrained eating. This theoretical model integrates three key psychological processes—behavioral control, emotional regulation, and stress coping, offering a comprehensive perspective on the mechanisms linking physical exercise to dietary behaviors.

**Figure 1:**
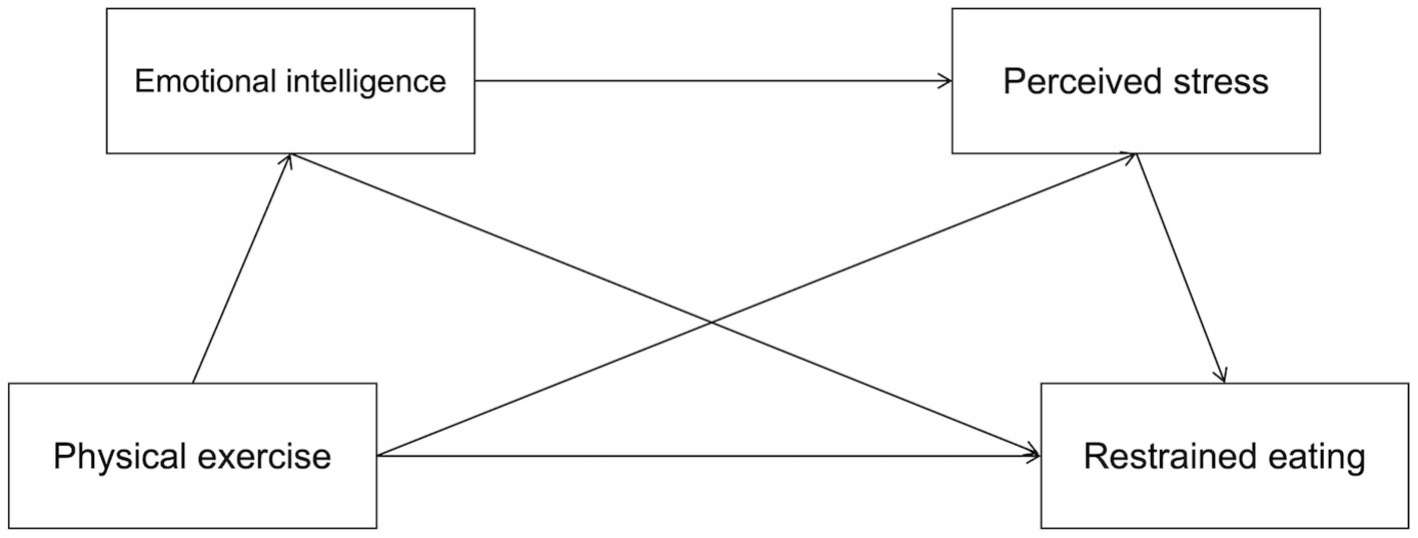
Chain-based mediation model diagram of physical exercise and restrained eating.

Based on the proposed conceptual framework (Figure 1), the following hypotheses are formulated:

1. Physical exercise is negatively associated with restrained eating.
2. Emotional intelligence mediates the relationship between physical exercise and restrained eating.
3. Perceived stress mediates the relationship between physical exercise and restrained eating.
4. Emotional intelligence and perceived stress jointly mediate the relationship between physical exercise andbreak restrained eating.

These hypotheses are grounded in the integrated theoretical framework outlined above. Testing this chain mediation model will contribute to a deeper understanding of the psychological mechanisms linking physical exercise to restrained eating among college students. The findings are expected to hold both theoretical and practical significance, offering insights for the development of targeted prevention and intervention strategies that address emotional intelligence and perceived stress in the context of eating behaviors.

## 3 Research methodology

### 3.1 Research sample

Data were collected using a self-administered questionnaire designed on the basis of existing literature and validated instruments. The questionnaire assessed college students' restrained eating behaviors, physical exercise habits, emotional intelligence, and perceived stress. Given that all participants were drawn from the Chinese population, the questionnaire was administered in Chinese. All scales employed have been previously translated and validated for use among Chinese college students, demonstrating satisfactory internal consistency and validity (Liang, 1994; Leung et al., 2010; Wu et al., 2017; Wang K. et al., 2020). The survey was conducted between October 23 and December 5, 2025, targeting college students from diverse regions of China. A stratified sampling method was employed. Universities were first stratified by geographic region (e.g., East China, South China, North China, Southwest China) and institution type (Project 985 universities, Project 211 universities, general comprehensive universities, sports-focused institutes, and polytechnic colleges). From each stratum, one to two universities were randomly selected, yielding a total of 12 participating institutions across eight provinces/municipalities: Beijing, Shanghai, Guangdong, Shandong, Sichuan, Shanxi, Jiangsu, and Hubei. Within each selected university, students were randomly recruited from various departments to enhance representativeness. Questionnaires were distributed online via the “Quanwenxing” platform under the supervision of trained research assistants. Prior to data collection, all assistants familiarized themselves with the questionnaire structure and completion instructions, and subsequently briefed participants on key considerations before they began responding. Participation was entirely voluntary. A total of 1,750 questionnaires were collected. To ensure data quality, the research team screened and excluded invalid responses based on the following criteria:

Inclusion criteria: (1) full-time enrollment in a Chinese university; (2) age 18 years or older; (3) provision of voluntary informed consent.

Exclusion criteria: (1) questionnaire completion time of less than 5 min (based on the median completion time of 9 min 10 s from the pilot study); (2) evidence of patterned responding (e.g., consecutive selection of the same option); (3) failure to correctly respond to an embedded attention check item (“Please select 2 or 4 for this question”).

After applying these criteria, 1,640 valid responses were retained, yielding a valid response rate of 93.71%.

### 3.2 Scale design

#### 3.2.1 Restrained eating

Restrained eating was assessed using the Restrained Eating subscale of the Dutch Eating Behavior Questionnaire (DEBQ), originally developed by Van Strien et al. (1986). This subscale consists of 10 items rated on a five-point Likert scale, with higher scores indicating more frequent engagement in restrained eating behaviors. In the present study, the subscale demonstrated excellent internal consistency, with a Cronbach's alpha coefficient of 0.934.

#### 3.2.2 Physical exercise

Physical exercise was assessed using the Physical Activity Rating Scale-3 (PARS-3), originally revised by Liang (1994) for use in Chinese populations. The scale measures participants' exercise behaviors over the preceding month across three dimensions: exercise intensity, exercise duration, and exercise frequency. Intensity and frequency are rated on 5-point scales, while duration is rated on a 4-point scale. Total exercise volume is calculated using the formula: Exercise volume = *Intensity* × *Duration* × *Frequency*, with higher scores reflecting greater levels of physical activity per session. In the present study, the scale demonstrated good internal consistency, with a Cronbach's alpha coefficient of 0.828.

#### 3.2.3 Emotional intelligence

Emotional intelligence was assessed using the Wong and Law Emotional Intelligence Scale (WLEIS), as revised by Law and colleagues (Law et al., 2004). The scale comprises 16 items rated on a 7-point Likert scale, measuring four dimensions: self-emotion appraisal, others' emotion appraisal, regulation of emotion, and use of emotion. Higher scores reflect greater emotional intelligence. In the present study, the scale demonstrated good internal consistency, with Cronbach's alpha coefficients of 0.884 for the total scale and 0.851, 0.819, 0.824, and 0.817 for the four subscales, respectively.

#### 3.2.4 Perceived stress

Perceived stress was measured using the Perceived Stress Scale (PSS), originally developed by Cohen and colleagues (Cohen et al., 1983). This unidimensional scale consists of 14 items, with items 4, 5, 6, 7, 9, 10, and 13 reverse-scored. Total scores are computed by summing all items, with higher scores indicating greater perceived stress. In the present study, the scale demonstrated excellent internal consistency, with a Cronbach's alpha coefficient of 0.953.

### 3.3 Statistical processing

This study employs quantitative analysis methods to examine the influence of physical exercise, emotional intelligence, and perceived stress on restrained eating behaviors among college students, based on cross-sectional survey data. All statistical analyses were performed using SPSS 26.0, the PROCESS macro for SPSS (Version 3.4; Model 6), and Excel 2021, following principles of transparency and reproducibility.

First, returned questionnaires were screened for validity. Of the 1,750 collected questionnaires, 1,640 were deemed valid, yielding a valid response rate of 93.71%. Second, common method bias was assessed using Harman's single-factor test. The results indicated that the first factor accounted for 29.009% of the total variance, below the recommended threshold of 40%, suggesting that common method bias was not a significant concern in this study (Aguirre-Urreta and Hu, 2019).

Descriptive statistics and Spearman's correlation coefficients were calculated for all study variables (physical exercise, restrained eating, emotional intelligence, and perceived stress). Hierarchical regression analysis was then conducted to examine the direct associations between these variables.

To test the proposed chain mediation model, the PROCESS macro (Model 6) was employed with 5,000 bootstrap samples to generate bias-corrected 95% confidence intervals for indirect effects. This procedure quantified the total, direct, and indirect effects of physical exercise on restrained eating through emotional intelligence and perceived stress, both independently and sequentially.

## 4 Results

### 4.1 Sample characteristics

A total of 1,640 valid questionnaires were obtained, yielding a valid response rate of 93.71%. Regarding residential background, 572 participants (34.88%) were from urban areas and 1,068 (65.12%) from rural areas. In terms of family composition, 447 participants (27.26%) were only children, while 1,193 (72.74%) had siblings. With respect to monthly living expenses, 51 participants (3.11%) reported expenditures below ¥500, 159 (9.70%) between ¥501 and ¥1,000, 665 (40.55%) between ¥1,001 and ¥1,500, 547 (33.35%) between ¥1,501 and ¥2,000, and 218 (13.29%) above ¥2,000. Regarding academic year, 561 participants (34.21%) were first-year undergraduates, 817 (49.82%) were second-year students, 198 (12.07%) were third-year students, and 64 (3.90%) were fourth-year students. The sample comprised 959 male students (58.48%) and 681 female students (41.52%). The mean age of participants was 19.29 years (*SD* = 1.16).

### 4.2 Common method bias test

Given the self-report nature of the data collection, common method bias was assessed using Harman's single-factor test. The results of an unrotated exploratory factor analysis revealed six factors with eigenvalues greater than one. The first factor accounted for 29.009% of the total variance, which is below the recommended threshold of 40% (Podsakoff et al., 2003). These results suggest that common method bias is unlikely to be a serious concern in the present study.

### 4.3 Descriptive statistics and correlations for each variable

Descriptive statistics and Spearman's correlation coefficients for all study variables are presented in Table 1. As shown, all variables were significantly correlated in the expected directions. Physical exercise was negatively associated with restrained eating (*r* = −0.369, *p* < 0.001) and perceived stress (*r* = −0.400, *p* < 0.001), and positively associated with emotional intelligence (*r* = 0.352, *p* < 0.001). Emotional intelligence was negatively associated with both restrained eating (*r* = −0.303, *p* < 0.001) and perceived stress (*r* = −0.238, *p* < 0.001). Perceived stress was positively associated with restrained eating (*r* = 0.275, *p* < 0.001). These significant correlations provide preliminary support for further mediation analysis.

**Table 1:** Descriptive statistics and correlation matrix for each variable.

Variable	Median	Quartile	Physical exercise	Restrained eating	Emotional intelligence	Perceived stress
Physical exercise	24	36 (12.48)	1			
Restrained eating	27	10 (22.32)	−0.369^***^	1		
Emotional intelligence	52	10 (48.58)	0.352^***^	−0.303^***^	1	
Perceived stress	38	13 (32.45)	−0.400^**^	0.275^***^	−0.238^***^	1

^**^indicates *p* < 0.01.

^***^indicates *p* < 0.001.

### 4.4 College students' physical exercise and restrained eating: a chain mediated effect

To examine the predictive relationships among physical exercise, emotional intelligence, perceived stress, and restrained eating, a regression analysis was conducted with restrained eating as the dependent variable and physical exercise, emotional intelligence, and perceived stress as independent variables. The results are presented in Table 2. Physical exercise was significantly negatively associated with restrained eating (β = −0.406, *t* = −17.957, *p* < 0.001), as was emotional intelligence (β = −0.337, *t* = −14.491, *p* < 0.001). In contrast, perceived stress was significantly positively associated with restrained eating (β = 0.332, *t* = 13.774, *p* < 0.001). These findings indicate that all three variables independently contribute to the prediction of restrained eating, with physical exercise and emotional intelligence serving as protective factors and perceived stress as a risk factor.

**Table 2:** Separate regression analyses of physical exercise, emotional intelligence, and perceived stress on restrained eating.

Variable	Restrained eating				
	β	* T*	*F*	*R* ^2^	*R* ^2^ *_*adj*_*
Physical exercise	−0.406	−17.957^***^	322.465	0.164	0.164^***^
Emotional intelligence	−0.337	−14.491^***^	209.979	0.114	0.113^***^
Perceived stress	0.332	13.774^***^	189.720	0.104	0.103^***^

*R*^2^_*adj*_ denotes adjusted *R*^2^;

^***^indicates *p* < 0.001.

The proposed chain mediation model was tested using the PROCESS macro for SPSS (Version 3.4, Model 6), with physical exercise as the independent variable (*X*), restrained eating as the dependent variable (*Y*), and emotional intelligence (*M*1) and perceived stress (*M*2) as sequential mediators. Bias-corrected bootstrap confidence intervals (95%) were generated using 5,000 resamples to assess the significance of indirect effects. A mediating effect was considered statistically significant if its confidence interval did not include zero (Sidhu et al., 2021).

**Figure 2:**
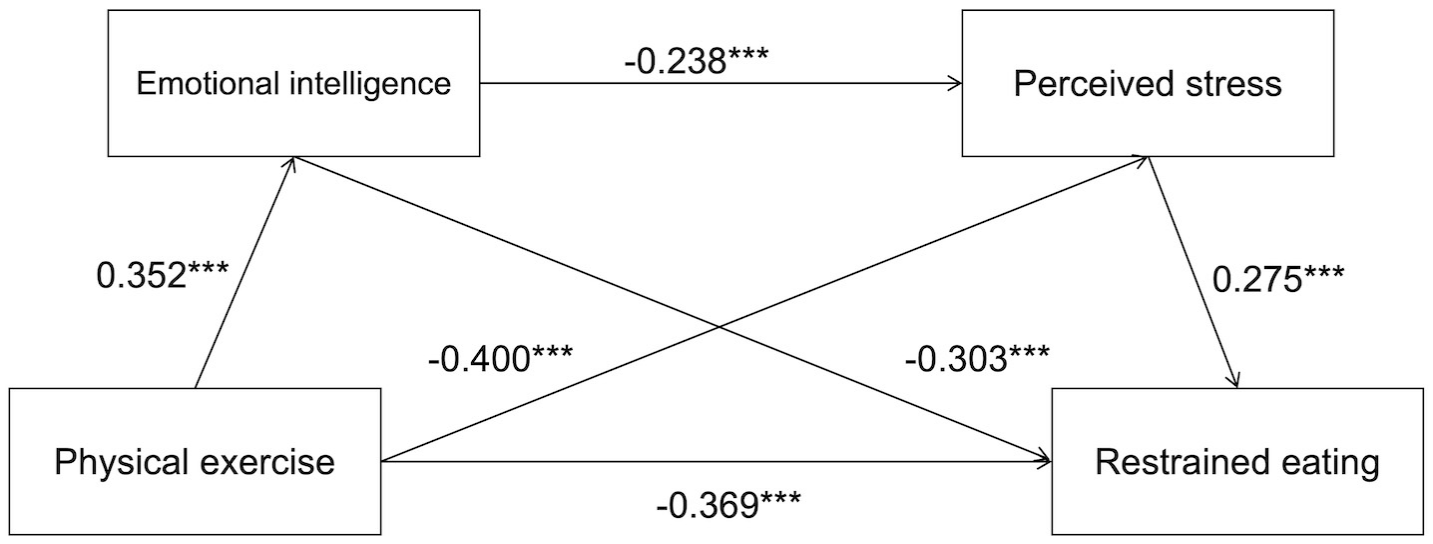
Path coefficient diagram of the final model. ***indicates *p* < 0.001.

The mediation results are presented in Table 3. Physical exercise had a significant total effect on restrained eating (β = −0.123, *p* < 0.001). The direct effect of physical exercise on restrained eating, after accounting for the mediators, remained significant (β = −0.079, *p* < 0.001, 95% *CI* [−0.136, −0.109]), supporting Hypothesis 1. Consistent with Hypothesis 2, emotional intelligence significantly mediated the relationship between physical exercise and restrained eating (indirect effect = −0.023, 95% *CI* [−0.030, −0.017]). Perceived stress also emerged as a significant mediator (indirect effect = −0.019, 95% *CI* [−0.025, −0.013]), confirming Hypothesis 3. Furthermore, the sequential mediating pathway through both emotional intelligence and perceived stress was significant (indirect effect = −0.002, 95% *CI* [−0.004, −0.001]), providing support for Hypothesis 4. The fact that all bootstrap confidence intervals excluded zero further supports the robustness of these mediating effects. Path coefficients for the full model are illustrated in Figure 2.

**Table 3:** Mediation effect size and effect magnitude.

Path	Effect	BOOT SE	BOOT LLCI	BOOT ULCI
Total effect	−0.123^***^	0.007	−0.136	−0.109
Direct effect	−0.079^***^	0.008	−0.094	−0.064
Total indirect effect	−0.044^***^	0.005	−0.054	−0.034
Indirect effect 1 (EI)	−0.023^***^	0.003	−0.030	−0.017
Indirect effect 2 (PS)	−0.019^***^	0.003	−0.025	−0.013
Indirect effect 3 (EI → PS)	−0.002^***^	0.001	−0.004	−0.001

Indirect effect 1: physical exercise → emotional intelligence → restrained eating.

Indirect effect 2: physical exercise → perceived stress → restrained eating.

Indirect effect 3: physical exercise → emotional intelligence → perceived stress → restrained eating.

^***^indicates *p* < 0.001.

Further effect analysis revealed that all three indirect effects were statistically significant, with a total effect size of −0.123. The direct effect of physical exercise on restrained eating was −0.079, and the total indirect effect via the two mediators was −0.044. Specifically, the indirect effect through emotional intelligence alone (Path 1: Physical Exercise → Emotional Intelligence → Restrained Eating) was −0.023, the indirect effect through perceived stress alone (Path 2: Physical Exercise → Perceived Stress → Restrained Eating) was −0.019, and the sequential indirect effect through both emotional intelligence and perceived stress (Path 3: Physical Exercise → Emotional Intelligence → Perceived Stress → Restrained Eating) was −0.002. Among these, emotional intelligence emerged as the strongest mediator. Taken together, these findings indicate that emotional intelligence and perceived stress partially mediate the relationship between physical exercise and restrained eating, with emotional intelligence playing a particularly prominent role in this chain mediating mechanism.

## 5 Discussion

### 5.1 The direct association between physical exercise and restrained eating

The findings of this study revealed a significant negative association between physical exercise and restrained eating among college students (*r* = −0.369). After controlling for relevant variables and incorporating mediators into the model, the direct association between physical exercise and restrained eating remained significant (β = −0.079, *p* < 0.001). While previous research has consistently demonstrated that higher levels of physical activity are associated with lower levels of dietary restriction, the present findings indicate that exercise alone explains only a modest proportion of the variance in restrained eating patterns. Thus, although the association is statistically significant, its practical magnitude warrants cautious interpretation. Nevertheless, these results support the perspective that physical activity may function as a protective health behavior, contributing to the prevention of maladaptive dietary patterns (Koreshe et al., 2023). This aligns with prior research suggesting that regular physical activity is associated with healthier eating attitudes and may is associated with lower the likelihood of developing eating disorders through both physiological and psychological mechanisms (Davis et al., 1994).

This finding indicates that physical exercise is not only positively associated with energy balance at the physiological level but may also offer individuals a more positive and sustainable approach to weight and health management at the behavioral level, thereby potentially decreasing their reliance on highly restrained eating strategies. In contrast to restrained eating, a high-risk and low-stability behavioral pathway, physical exercise represents a more adaptive health choice for college students, thereby mitigating adverse eating behaviors that may arise from weight- or appearance-related concerns. The observed correlation (*r* = −0.369) reflects a moderate association between physical exercise and restrained eating. After accounting for the mediating variables, the direct effect remained significant yet small in magnitude (β = −0.079), suggesting that additional mechanisms beyond emotional intelligence and perceived stress may also contribute to this relationship.

### 5.2 The mediating effect of emotional intelligence

Mediation analysis revealed that emotional intelligence significantly mediated the relationship between physical exercise and restrained eating, with an indirect effect of −0.023, the largest among the three mediating pathways. Although this indirect effect is considered small in absolute magnitude according to conventional guidelines (Cohen et al., 1983), its relative contribution, accounting for 52.3% of the total indirect effect, underscores the pivotal role of emotional intelligence in explaining the association between physical exercise and restrained eating. This finding suggests that emotional intelligence serves as a key psychological mechanism through which physical exercise is associated with restrained eating in this population.

Previous research has demonstrated that physical exercise is positively associated with emotional intelligence, which in turn is closely linked to healthier behavioral choices (Wang S. B. et al., 2020). The present study extends this evidence by showing that physical exercise is not only directly associated with dietary behavior but may also is associated with lower college students' tendency to engage in restrained eating as a means of emotional regulation, specifically by enhancing their capacity to recognize, understand, and regulate emotions.

Within the context of Chinese society and culture, this finding may be closely related to the competitive pressures faced by college students in urbanized environments and the collective living conditions inherent in university life (Saklofske et al., 2012; Chen et al., 2022). In highly socialized learning and living environments, high levels of emotional intelligence enable individuals to manage interpersonal interactions and psychological stress more effectively, thereby playing a more central role in facilitating healthy behavioral choices. This interpretation is consistent with recent empirical evidence indicating that, among Chinese young adults, higher perceived stress was associated with unhealthy eating patterns only among individuals with poorer emotional regulation, highlighting the buffering role of emotional competence in the stress-eating relationship (Chen et al., 2024). Furthermore, cross-cultural research suggests that the understanding and application of emotional intelligence must be considered within cultural context; collectivist societies may place different emphases on emotional expression and regulation compared to individualist cultures (Walter et al., 2024), which may explain the prominent mediating role of emotional intelligence observed in our Chinese sample.

This finding corroborates existing research highlighting the pivotal role of emotional intelligence in college students' psychological adaptation and behavioral selection (Samadiyeva et al., 2025). For instance, research has indicated that team-based sporting activities typically involve more frequent emotional expression, social interaction, and cooperative scenarios, thereby aiding individuals in developing their capacity for emotional perception, comprehension, and regulation within authentic contexts (Batinić et al., 2014). On this basis, the present study suggests that within university physical activity programs, team-based sports characterized by social interaction may offer greater advantages compared to solitary or isolated forms of exercise in fostering emotional intelligence development and thereby reducing the risk of restrained eating behaviors.

### 5.3 The mediating effect of perceived stress

The present findings indicate that perceived stress significantly mediates the relationship between physical exercise and restrained eating. This result aligns with stress-related eating behavior models, which posit that heightened stress levels are associated with increased vulnerability to restrictive or disordered eating patterns as a means of regaining control or alleviating negative emotions (Fox and Power, 2009).

Physical exercise has been widely recognized as an effective strategy for alleviating stress, and reducing perceived stress represents a key pathway through which physical activity promotes healthier dietary behaviors among college students (Jiazhi et al., 2025). When individuals experience lower levels of perceived stress, their need to restore a sense of control or mitigate negative emotions through restrained eating diminishes accordingly, thereby reducing the likelihood of engaging in such behaviors. The indirect effect of perceived stress (β = −0.019) was small yet meaningful, accounting for 43.2% of the total indirect effect. This finding supports stress reduction as a relevant pathway linking physical exercise to lower levels of restrained eating.

### 5.4 The chain mediating effect of emotional intelligence and perceived stress

Chain mediation analysis revealed that emotional intelligence and perceived stress form a significant sequential mediating pathway between physical exercise and restrained eating. This finding suggests that physical exercise may be indirectly associated with college students' restrained eating behaviors through a sequential mechanism involving enhanced emotional intelligence and subsequently is associated with lowered perceived stress.

This finding offers a more integrated perspective on the complex psychological mechanisms through which physical exercise is associated with dietary behavior. Enhanced emotional intelligence facilitates more adaptive appraisal and management of stress, while is associated with lowered perceived stress further diminishes reliance on restrained eating as a compensatory control strategy. This chain mediation aligns with theoretical frameworks emphasizing the interactive roles of emotional intelligence and perceived stress in shaping health behaviors (Kadović et al., 2022). It also provides a foundation for future research to explore the psychological effects of physical exercise from a dynamic and process-oriented perspective.

It is worth noting that although all indirect effects were statistically significant, the effect sizes were relatively small. Several factors may account for this pattern. First, in mediation models involving sequential pathways, the product of multiple path coefficients inevitably yields a value smaller than the direct effect or a single mediating effect (Kenny and Judd, 2014). Second, the total indirect effect (−0.044) accounted for 35.8% of the total effect (−0.123), indicating that emotional intelligence and perceived stress jointly made a meaningful contribution to explaining the association between physical exercise and restrained eating. Third, from a public health perspective, even small effects can carry substantial significance when applied to large populations. Given that weight concerns and dietary control are highly prevalent among college students, interventions targeting emotional intelligence and stress management—even those with modest individual-level effects—may still yield considerable population-level benefits. Future research should explore additional mediating variables, such as body image flexibility and self-efficacy, to further account for the remaining variance. Taken together, these findings provide empirical support for the theoretical framework outlined above, demonstrating that physical exercise is associated with restrained eating through sequential pathways involving emotional intelligence (Emotion Regulation Theory) and perceived stress (Stress and Coping Theory).

### 5.5 Research limitations and future prospects

This study has several limitations that should be acknowledged. First, the cross-sectional design precludes causal inferences from the observed associations; the directional paths in our model are based on theoretical assumptions rather than empirical causation. Future research should validate the proposed model using longitudinal designs or experimental interventions. Second, all variables were measured using self-report instruments, which may be susceptible to recall bias and social desirability effects (Caputo, 2017). Future studies could incorporate objective measures of physical activity or multi-informant assessments to enhance measurement validity. Third, although the sample included students from diverse university types across multiple regions in China, the findings may not be fully generalizable to non-student populations or to individuals from other cultural backgrounds. Moreover, the cross-sectional design cannot test reverse causal relationships. While our theoretical model assumes that physical exercise influences restrained eating, the opposite direction may also hold: individuals with higher levels of restrained eating may is related to physical activity due to insufficient energy intake (Forney et al., 2025), or they may increase exercise engagement due to heightened weight concerns (Davis et al., 1994). Future research should employ longitudinal or cross-lagged panel designs to examine these bidirectional relationships.

In addition, the sample had a higher proportion of male participants (58.48%), and no gender-group analysis was conducted. Given that restrained eating is more prevalent among females (Welch et al., 2015) and that emotional intelligence and perceived stress may vary by gender, future studies should employ gender-balanced samples and test whether gender moderates the proposed chain mediation model. Furthermore, age and body mass index were not controlled for in the mediation analyses. Although our primary focus was on psychological mechanisms, these demographic and physiological variables may be associated with the observed relationships (Welch et al., 2015; Yong et al., 2021). Future research should include them as covariates to improve the precision of estimated associations.

Additionally, the present study did not differentiate between types of physical activity or levels of athletic competition, factors that may influence the relationship between exercise and eating behaviors. Future research should examine these dimensions more closely. Meriç and Yabanci Ayhan (2025) found that intuitive and mindful eating are negatively associated with BMI and eating disorder symptoms, suggesting that promoting adaptive eating styles may represent a promising alternative. Future research should investigate whether physical exercise enhances not only emotional intelligence but also intuitive eating, and whether these factors jointly contribute to healthier eating patterns.

Despite these limitations, this study enriches empirical research in the field by systematically elucidating the mechanisms through which physical exercise, emotional intelligence, and perceived stress influence restrained eating behaviors among college students. Findings indicate that promoting physical exercise not only holds direct health benefits but may also indirectly is associated with lower restrained eating behaviors by enhancing emotional intelligence and reducing the perceived stress. This discovery offers practical implications for higher education institutions in designing physical education programmes and mental health education. Integrating physical exercise with emotional competence development and stress management training may represent an effective pathway for intervening in unhealthy eating behaviors among college students.

## 6 Conclusion

This study systematically examined the relationship between physical exercise and restrained eating among Chinese college students, with a particular focus on the mediating roles of emotional intelligence and perceived stress. The findings revealed that physical exercise is significantly negatively associated with restrained eating, and that emotional intelligence and perceived stress partially mediate this relationship through a significant chain mediation pathway.

Specifically, physical exercise was found to be not only directly associated with lower levels of restrained eating but also indirectly linked through its positive association with emotional intelligence and its negative association with perceived stress. Emotional intelligence emerged as the strongest mediator among the indirect pathways, underscoring its central role in this process. Perceived stress, in turn, acted as a risk-amplifying factor, further elucidating the psychological mechanisms through which physical exercise relates to dietary behavior. The chain mediation analysis further demonstrated that physical exercise may influence college students' eating behaviors through a sequential process involving enhanced emotional competence and subsequently is associated with lowered perceived stress.

By integrating behavioral and psychological perspectives, this study extends the existing research framework on the relationship between physical exercise and dietary behavior, contributing to a deeper understanding of the mechanisms underlying restrained eating. On a practical level, the findings suggest that higher education institutions should consider the multifaceted role of physical exercise in promoting student health. Interventions that combine physical activity with emotional intelligence training and stress management, particularly those that encourage participation in group-based sports, may be especially effective in reducing restrained eating behaviors and supporting the holistic well-being of college students.

## Data Availability

The original contributions presented in the study are included in the article/supplementary material, further inquiries can be directed to the corresponding author.
